# Nutrient reference value: non-communicable disease endpoints—a conference report

**DOI:** 10.1007/s00394-016-1195-z

**Published:** 2016-03-16

**Authors:** J. R. Lupton, J. B. Blumberg, M. L’Abbe, M. LeDoux, H. B. Rice, C. von Schacky, A. Yaktine, J. C. Griffiths

**Affiliations:** Emeritus, Texas A&M University, College Station, TX USA; Tufts University, Boston, MA USA; University of Toronto, Toronto, ON Canada; Natural Alternatives International, Inc., San Marcos, CA USA; Global Organization for EPA and DHA Omega-3s, Salt Lake City, UT USA; University of Munich, Munich, Germany; The National Academies of Sciences, Engineering, and Medicine, Washington, DC USA; Council for Responsible Nutrition, Washington, DC USA

**Keywords:** Bioactives, Nutrient reference values, nonessential nutrients, Adequate intake, n-3 long-chain polyunsaturated fatty acids, Omega-3 fatty acids, Docosahexaenoic acid (DHA), Eicosapentaenoic acid (EPA)

## Abstract

Nutrition is complex—and seemingly getting more complicated. Most consumers are familiar with “essential nutrients,” e.g., vitamins and minerals, and more recently protein and important amino acids. These essential nutrients have nutrient reference values, referred to as dietary reference intakes (DRIs) developed by consensus committees of scientific experts convened by the Institute of Medicine of the National Academy of Sciences, Engineering, and Medicine and carried out by the Food and Nutrition Board. The DRIs comprise a set of four nutrient-based reverence values, the estimated average requirements, the recommended dietary allowances (RDAs), the adequate intakes and the tolerable upper intake levels for micronutrient intakes and an acceptable macronutrient distribution range for macronutrient intakes. From the RDA, the US Food and Drug Administration (FDA) derives a labeling value called the daily value (DV), which appears on the nutrition label of all foods for sale in the US. The DRI reports do not make recommendations about whether the DV labeling values can be set only for what have been defined to date as “essential nutrients.” For example, the FDA set a labeling value for “dietary fiber” without having the DV. Nutrient reference values—requirements are set by Codex Alimentarius for essential nutrients, and regulatory bodies in many countries use these Codex values in setting national policy for recommended dietary intakes. However, the focus of this conference is not on essential nutrients, but on the “nonessential nutrients,” also termed dietary bioactive components. They can be defined as “Constituents in foods or dietary supplements, other than those needed to meet basic human nutritional needs, which are responsible for changes in health status (Office of Disease Prevention and Health Promotion, Office of Public Health and Science, Department of Health and Human Services in Fed Regist 69:55821–55822, 2004).” Substantial and often persuasive scientific evidence does exist to confirm a relationship between the intake of a specific bioactive constituent and enhanced health conditions or reduced risk of a chronic disease. Further, research on the putative mechanisms of action of various classes of bioactives is supported by national and pan-national government agencies, and academic institutions, as well as functional food and dietary supplement manufacturers. Consumers are becoming educated and are seeking to purchase products containing bioactives, yet there is no evaluative process in place to let the public know how strong the science is behind the benefits or the quantitative amounts needed to achieve these beneficial health effects or to avoid exceeding the upper level (UL). When one lacks an essential nutrient, overt deficiency with concomitant physiological determents and eventually death are expected. The absence of bioactive substances from the diet results in suboptimal health, e.g., poor cellular and/or physiological function, which is relative and not absolute. Regrettably at this time, there is no DRI process to evaluate bioactives, although a recent workshop convened by the National Institutes of Health (Options for Consideration of Chronic Disease Endpoints for Dietary Reference Intakes (DRIs); March 10–11, 2015; http://health.gov/dietaryguidelines/dri/) did explore the process to develop DVs for nutrients, the lack of which result in increased risk of chronic disease (non-communicable disease) endpoints. A final report is expected soon. This conference (CRN-International Scientific Symposium; “Nutrient Reference Value—Non-Communicable Disease (NRV-NCD) Endpoints,” 20 November in Kronberg, Germany; http://www.crn-i.ch/2015symposium/) explores concepts related to the Codex NRV process, the public health opportunities in setting NRVs for bioactive constituents, and further research and details on the specific class of bioactives, n-3 long-chain polyunsaturated fatty acids (also termed omega-3 fatty acids) and their constituents, specifically docosahexaenoic acid and eicosapentaenoic acid.

## Introduction

This scientific symposium, to which these proceedings contribute this conference report, again ask the question “Is it important to have a dietary reference intake- (DRI-) like [also termed nutrient reference value- (NRV-) like] process for the evaluation of bioactives?” Research continues to suggest that there are real benefits to human public health to be obtained from including bioactive constituents in the diet and/or from dietary supplements. Past symposia have looked at common bioactives, including flavanols from tea and chocolate; lycopene and other tomato carotenoids; lutein and zeaxanthin and soybean isoflavones isolated from plants and animals (fish oil) [3, 39]. The wide range of academic institutions, from virtually every part of the globe, confirm the seriousness to which researchers are exploring the biochemical and biophysiological processes attributed to these substances.

Consumers deserve clarity and transparency regarding bioactive content and access to reliable information on products. But how is the public able to understand the strength (and/or limitations) of the science supporting the putative benefits—and even if the link between intake and disease risk reduction is confirmed and advertised and accepted, how much is the right amount to consume? Too little and the desired effect might not be realized—too much and overt toxicity may develop. An evaluative process was proposed 2 years ago at this same scientific symposium (see Table 1), and it continues to be discussed and evaluated as a framework approach.

**Table 1:** Why it is important to have a DRI-like process for the evaluation of *bioactives* [39]

Importance	Example	Benefit for having a DRI-like value
Bioactives are important to human health	For example, there is strong science behind the relationship between flavanols and decreased risk of cardiovascular disease [18, 27]; isoflavones and lower risk of several chronic diseases [31, 60, 68]; and lycopene and other tomato carotenoids and decreased blood pressure [6, 15, 28, 50]	A major benefit would be that they would be recognized as being important to health and evaluated accordingly. Investigators, regulatory agencies, consumers would all know how strong the science was behind science messaging on these compounds
Bioactives are a significant portion of diet and disease research portfolios	Governments, universities, and food manufacturers are supporting studies on bioactives	Standards would be set so that studies could be compared across laboratories
Consumers are interested in optimal health and are purposefully purchasing foods containing bioactives	This was part of the rationale for setting DRI values for bioactives in China	Consumers would benefit from strengthened knowledge that they were making decisions based on science and they would also have a target to aim for in terms of intake
Having a DRI value elevates the status of a bioactive and makes it part of nutrition public policy	Substances that have DRI values are regularly evaluated in populations to see whether that population is meeting established DRI values	If the bioactive is part of the intake assessment of nutrients/foods, then we will learn whether or not that population is actually meeting the DRI value, or if it is an “at risk” nutrient
The process by which a bioactive is evaluated would set standards which would raise the level of science	Such requirements as having a formal definition, and an approved method of analysis would help comparing studies across laboratories	Using common methods of analysis and a common definition would allow studies to build on each other and advance the science more rapidly
With a transparent process for evaluation the results would provide science-based recommendations for improving diets	Health professionals such as doctors, dietitians, and educators would be more comfortable making diet recommendations	Messaging on intake of bioactives would be science-based
Having an intake value would set a goal for incorporating bioactives into diets	Consumers would know whether a food was a good source of that bioactive, or how much one would need to eat in order to reach the intake value	Having a target intake value would discourage messaging on products that suggest they are a good source of a specific bioactive when they only contain a negligible amount

If the science base was translated into a recommended intake value, assessments could be made as to whether or not populations or specific age groups were meeting that recommendation and consumers would know the overall contribution of a food or beverage or dietary supplement to achieving the recommended amount.

If an authoritative body such as the Institute of Medicine IOM or the Codex Alimentarius Commission would establish reference values for bioactives that promote health or contribute to a reduction in risk of non-communicable disease endpoint (NRV-NCD), then the bioactive of merit would become part of national and international nutrition policy and be used to bolster public health initiatives. For example, in the US, with a realistic DRI value, intake information as incorporated into and referenced by the National Health and Nutrition Examination Survey (NHANES) would allow the government agencies and researchers to understand the amount of nutrient(s) being consumed and be able to address “at risk” low nutrient (bioactive) consumers. Having a DRI, or even just an element such as the adequate intake (AI), would assist responsible authorities in developing education and messaging to the consumers at risk or at borderline for the health-promoting, disease-reducing benefits of specific bioactives. It is the stated objective of the US Dietary Guidelines for Americans that if one follows all of the recommendations of the guidelines, one will automatically meet the DRI values for nearly all of the nutrients [45]. Thus, dietary guidance is another important way that information on bioactives with substantial science behind their efficacy could be transmitted to consumers.

In the twenty-first century, advances in research of health-giving nutrients is demonstrating promise in achieving reductions in morbidity and mortality in formerly high-risk populations. What regulators do with this research is of paramount importance. The time for parochialism and market protectionism thinly disguised as bona fide barriers to commercial trade based on unsound reasoning are over. The regulatory and scientific communities need to revisit their historical biases in favor of a more rational outcome objective.

## Setting an intake value for bioactives

There are multiple reasons why a framework for the evaluation of bioactives should be considered. The primary reason is to promote public health (i.e., “What is an efficacious amount?”; “How strong is the science?”; “What are potential adverse effects?”). Would setting an intake value for a bioactive fit the DRI paradigm?

Food is now viewed by many as a source of substances that provide robust cell/tissue functions or optimal health rather than just to protect against nutrient deficiency diseases. It is important to provide consumers with information as to how strong the science is behind purported benefits, how much they would need to eat to achieve these benefits, and how much is too much.

What are the major issues involved in setting an intake value for bioactives? First, bioactives are different from essential nutrients in that the absence of the bioactive in the diet does not result in a deficiency disease specific to that bioactive. In contrast, if an essential nutrient is absent from the diet one will get a deficiency—microcytic anemia for lack of iron, scurvy for vitamin C, beriberi for thiamin, etc. This means that an intake value for a bioactive cannot be based on a deficiency disease; instead, alternative endpoints for intake values are needed. Some have suggested basing intake values on endpoints characterizing “health” rather than lack of disease, which is philosophically attractive, but this is still a developing science base. Another type of endpoint, appropriate for bioactives might be a beneficial physiological effect such as blood pressure or vascular reactivity. This is an important area of research but does not have much traction in the US. A third alternative is basing the endpoint on decreased risk of disease. This has elicited some traction for the US/Canada DRI Government subcommittee for DRIs. Also, much of the research done with bioactives is targeted at decreased risk of non-communicable diseases (NCDs). In fact, using chronic disease endpoints to determine intake values was a major recommendation coming from two key IOM publications [25, 65]. In the process, an intake value (AI) was developed and used as the intake value for several nutrients. The AI is defined as “The recommended average daily intake level based on observed or experimentally determined approximations or estimates of nutrient intake by a group (or groups) of apparently healthy people that are assumed to be adequate—used when a recommended dietary allowance (RDA) cannot be determined.” If an intake value for a bioactive were to be based on a chronic disease endpoint, it would likely be an AI rather than an RDA. In part this is because the absolute risk of most chronic diseases applies to only a portion of the population, rather than the entire population for a deficiency disease which is nutrient-specific (e.g., scurvy for vitamin C). Chronic diseases are not nutrient specific, but multifactorial.

## The DRI process: strengths and limitations of evidence

The process of setting standardized nutrient intake values was formalized in the US more than 75 years ago in response to widespread nutrient deficiency diseases in the general population. Following establishment of the RDAs, the prevalence of frank nutrient deficiency disease diminished; however, in the subsequent decades, risk of a range of diet-related chronic diseases increased. Cardiovascular disease, including stroke and coronary artery disease, obesity, type 2 diabetes, and cancer are now predominant public health concerns. In response to the 1988 Surgeon General’s Report [62], the 1989 Diet and Health Report [46], and a 1994 IOM Report [23], reduction in risk of chronic disease, identification of food components related to health benefits, and evaluation of risk from both deficiency and excess were identified as relevant concerns to be addressed in revising the RDAs. The outcome was a framework for what would become the DRIs. The new paradigm was based on reducing both the risk of inadequacy of nutrient intakes and the risk of adverse outcomes from excessive intakes using a risk assessment model. Today, the goal is to understand how the DRI paradigm can be applied to identification of nutrients and other food components to achieve reduction in relative risk of diet-related chronic disease. A number of factors limit use of the current DRI process to identify intake levels sufficient for chronic disease risk reduction at the population level. Major barriers include: population variability in disease risk, lack of dose–response relationships for health outcomes, identifying upper level intake thresholds, and insufficient evidence to support setting DRIs for nutrients with chronic disease endpoints. For five nutrients identified for DRIs, limitations in available evidence for reduction of chronic disease risk led to an AI rather than an RDA across all age categories. The challenge moving forward is to determine whether or how the DRI process can be adapted to nutrients such as the “nonessential” nutrients, EPA and DHA found in omega-3 fatty acids that have implications for reduction in risk of chronic disease. That will require consideration of important research gaps, including biomarker validation studies, determining safe and effective upper and lower nutrient intake levels, nutrient interactions with other substances in foods, and determination of nutrient requirements across age groups.

## Evidence-based nutrition: the problem of proof

Over the last decade, randomized clinical trials (RCTs) of dietary supplements and nutritional interventions for major disease entities have largely resulted in null or negative outcomes despite positive results from in vitro, animal model, and observational studies. Because RCTs have traditionally been accepted as the “gold standard” for establishing cause-and-effect relationships, these studies have led to skepticism about the importance of specific nutrients or nutrient combinations in health and disease by clinicians, researchers, funding agencies, and the public. Nonetheless, the foundation of RCTs in evidence-based medicine has now been wholly adopted in the creation of nutrition and science policy despite distinct differences between the evidence needed for testing of drugs versus that needed for the development of nutrient requirements and dietary guidance. There is a need to better define the types of evidence necessary for developing dietary guidelines and recommending nutrient interventions than that used for drug efficacy and safety. For example, unlike drugs, nutrients and other dietary bioactive components work in complex networks, are often under homeostatic control, and cannot be contrasted to a true placebo group. Although RCTs present one approach toward understanding the efficacy of nutrient interventions, the innate complexities of nutrient actions and interactions cannot always be adequately addressed through a single research design. Further, action to define requirements for nutrients and dietary bioactive components or to recommend dietary guidelines to promote health and/or reduce the risk of chronic disease should be taken at a level of confidence that is different from that needed in the evaluation of drug efficacy and safety in the treatment of disease. Moreover, in assessing the balance between the potential harm of making or not making a nutrient or dietary recommendation, appropriate educational strategies will be necessary to convey the varying levels of the strength of the evidence. Advancing evidence-based nutrition from its current version to one based upon more relevant and realistic criteria will depend upon research approaches that include RCTs but go beyond them [4, 55, 56].

## Omega-3 fatty acid (EPA/DHA) research

Fatty acids are necessary components of cell membranes, and changes in fatty acid composition of these membranes can modulate cell function. While some fatty acids, like saturated or some mono-unsaturated fatty acids can be formed by the human body, others, like the omega-3 fatty acid alpha-linolenic or the omega-6 fatty acid linoleic, cannot. It was thought that longer chain polyunsaturated fatty acids (PUFAs) of both the omega-3 and omega-6 type could be formed in humans from the shorter chain fatty acids mentioned. This, however, does not occur in quantities sufficient to maintain optimal function of the respective cells (e.g., brain, heart) [7, 51]. Therefore, some PUFAs, especially eicosapentaenoic acid (EPA) or docosahexaenoic acid (DHA), need to be obtained from the diet.

Diet was the traditional focus of research in nutrition. However, results obtained with food frequency questionnaires were recently found to be physiologically implausible and their validity thus extremely limited [2]. Moreover, fatty acid compositions of dietary components change, and fatty acid uptake from the gut and incorporation into cells varies inter-individually by a factor of 13 [30]. To circumvent these three levels of uncertainty, erythrocyte EPA + DHA is being used as a biomarker for marine omega-3 fatty acids [64]. Erythrocyte EPA + DHA has a low biological variability and correlates with EPA + DHA in all cell types studied so far, and a standardized analytical procedure (HS-Omega-3 Index^®^) is available, which is the basis of some 174 publications international journals and >50 ongoing research projects, some of which are considered below [29, 35, 63, 64, 71].

In Western countries, including Canada or Germany, diseases like cardiovascular disease, major depression, or cognitive impairment have a high prevalence, and more than three quarters of the population have an HS-Omega-3^®^ Index below the target healthy range of 8–11 % [35, 64]. By current standards of the American Heart Association, low levels of EPA + DHA in erythrocytes are a cardiovascular risk factor [64]. Due to issues in methodology uncovered by measuring EPA + DHA in erythrocytes, pertinent large intervention trials had neither positive nor negative, i.e., null outcomes [64]. Because this fact hampers widespread use of EPA + DHA in cardiovascular prevention, a new generation of large trials with clinical endpoints is needed.

Low levels of erythrocyte EPA + DHA have been found in individuals with suboptimal brain development, attention deficit-hyperkinetic disorder, major depression, or issues in complex brain function, like memory, executive function and others in all age groups studied so far. In all of these impairments of cognitive function, most randomized controlled intervention trials demonstrated positive effects of EPA + DHA supplementation, quantitatively correlating with the increase of EPA + DHA in erythrocytes, if measured. Thus, a causal role of EPA + DHA has been identified in the brain function issues mentioned. Moreover, consistent with observational studies showing EPA + DHA intake or status at suboptimal levels in Western countries, these data support the interpretation that, in Western countries (but not in countries like Japan or Korea), a widespread deficit of EPA + DHA intake exists.

Other health issues for which positive effects of increased intake or an elevation of erythrocyte EPA + DHA have been demonstrated are muscle function, osteoporosis, rheumatoid arthritis, non-alcoholic fatty liver, and others. Of note, “age-related” deterioration of brain and muscle function can be slowed by EPA + DHA, again supporting the interpretation of a widespread deficit in Western countries [57, 67].

## Scientific basis for a public health recommendation for EPA/DHA

Globally, consumption of the omega-3 fatty acids, EPA and DHA is inadequate for cardiovascular disease risk reduction. In 2010, the attributable burden of a diet low in seafood omega-3s (rich in EPA and DHA) was 1.1 % of global disability-adjusted life years (DALYs), a measure of overall disease burden, expressed as the number of years lost due to ill health, disability, or early death [38]. That same year, diets low in EPA + DHA accounted for 1,389,896 deaths, up from 1,043,085 in 1990 [38]. Given the recent commencement of work to establish a NRV-NCD for EPA + DHA, the totality of the scientific evidence supporting the cardiovascular benefits of EPA/DHA is important to consider [9].

There is a long history of research demonstrating that EPA and DHA have cardioprotective benefits. This research includes both observational studies and RCTs. In contrast to earlier investigations [5, 21, 70]; GISSI-HF Investigators, [20] demonstrating that consumption of fatty fish or EPA/DHA supplements have clear benefits for cardiovascular health, some recent studies [19, 33, 40]; ORIGIN [49, 52, 53, 59] have not demonstrated significant effects of EPA and DHA on cardiovascular disease (CVD) risk/events.

Potential reasons why recent research has resulted in null outcomes include, but are not limited to, the following: insufficient dose of omega-3s, treatment duration too short, maintenance on aggressive cardiovascular drug treatment, too few subjects, use of composite endpoints, higher background omega-3 intake, and subjects with such advanced CVD that you wouldn’t expect a benefit at such a late stage of the disease [22, 26, 41, 64, 69]. Given the preponderance of positive research in the past, the recent null results may be considered anomalies. The reality is that the evidence for many outcomes is very consistent.

For example, the evidence that EPA/DHA reduce the risk of cardiac death is strong. Between 2008 and 2014, 11 meta-analyses were published on the cardiovascular benefits of EPA/DHA. Each of the 11 meta-analyses consistently demonstrated a statistically, not to mention clinically, significant reduction (9–32 %) in the risk for cardiac death [8, 10, 12, 32, 34, 37, 42, 54, 61, 66, 72].

In addition to the consistency of results associated with EPA/DHA and cardiac death risk reduction across a wide range of doses, EPA/DHA consistently provide a statistically significant reduction in blood pressure, a biomarker for coronary heart disease. In 2014, a comprehensive meta-analysis of 70 RCTs on the effects of EPA and DHA (from seafood, fortified foods, or dietary supplements) on blood pressure was published [43]. The study included trials with subjects with normal blood pressure and those with hypertension but not taking blood pressure-lowering medications. Among all subjects, the average decrease in systolic blood pressure (SBP) and diastolic blood pressure (DBP) was 1.52 and 0.99 mm Hg, respectively. Among subjects with high blood pressure, the average decrease in SBP and DBP was 4.51 and 3.05 mm Hg, respectively. The findings were considered even more dramatic when compared to reductions achieved through commonly recommended lifestyle changes like reducing intake of dietary sodium (3.6 mm Hg), increasing physical activity (4.6 mm Hg), and decreasing alcohol consumption (3.8 mm Hg) [13].

To establish a NRV, it is important to consider not only the efficacy, but the safety of the nutrient(s) in question. According to the Codex Guidelines on Nutrition Labelling, the establishment of general population NRVs should take into account upper levels (ULs) established by the Food and Agriculture Organization of the United Nations (FAO) and the World Health Organization (WHO) or other recognized authoritative scientific bodies [11]. For over 25 years, every known comprehensive safety evaluation on EPA/DHA has concluded that there is insufficient evidence to establish an UL for EPA/DHA because of a lack of observed untoward outcomes [24, 44]; Norwegian Scientific Committee for Food Safety, [14, 47].

In the absence of a UL, FAO/WHO introduced the highest observed intake (HOI) level [16]. “The HOI is derived only when no adverse health effects have been identified. It is the highest level of intake observed or administered as reported within (a) study(ies) of acceptable quality.” In order to establish a NRV for EPA + DHA, the concept of a HOI will need to be accepted by Codex. Recent safety evaluations [14, 47] concluding the absence of sufficient evidence to establish an UL for EPA + DHA noted no safety concerns with levels from 5.0 to 6.9 g per day—levels at least 20× higher than the FAO minimum recommended intake of 250 mg/day [17].

## Summary, conclusion, and next steps

Each bioactive is unique, and the scientific underpinnings to the strength (breadth and depth) of scientific data vary. Some bioactives benefit from many decades of research in support of a purported reduction in disease risk. Others are overnight sensations, coming from traditional medicines in developing countries and regions. A framework approach must be able to differentiate and apply a tiered approach to the evaluation. Dr Lupton has proposed that there exists, “a high standard for entrance into the evaluative process.” Two years ago at this same scientific symposium, Dr. Lupton discussed potential entrance criteria as necessary information before a *bioactive* could be considered for a DRI-like evaluation process (see Table 2). Setting these nine criteria as essential for consideration for evaluation serves several goals: It minimizes the effort of the evaluator; and importantly, it sets a standard, if met, that investigators and funding sources could design their research to meet, knowing that there would be a certain level of credibility if they were to do so.

**Table 2:** Proposed criteria for a bioactive to qualify for evaluation [39]

Criterion	Additional information	Rationale for criterion
A definition of the substance which is commonly accepted	Definition should match the method of analysis	Makes it easier to build a database of efficacy of bioactive if substances with the same definition are compared
A method of analyzing the substance which is consistent with the definition	Preferably backed up by a multicenter analysis such as an AOAC method	Facilitates comparing studies across laboratories. Need a definition and an approved method of measuring so that intake values can be determined, and if populations are meeting recommended intake values
Database of the amount of the bioactive in foods	Preferably global and updated on a regular basis as new foods come on the market	To determine the amount of this bioactive currently in the food supply and enable determining how much people are consuming. Also necessary for baseline data for clinical trials and input into epidemiological studies
Prospective cohort studies	Both sexes, showing decreased risk of a disease such as CVD with increased intake of the bioactive. Must be able to isolate the specific bioactive vs other bioactives. Best if the bioactive is also measured in blood/urine, etc., in subset of population and supports food intake data. Relationship to the disease should be consistent with clinical trials	Dose response data or at least highest quintile vs lowest quintile for the bioactive will help to set level of efficacy
Clinical trials on digestion, absorption, activation, transport, excretion of the substance	Important to understand the level of absorption and what substances interfere with that absorption, also what the active molecule is and how long it stays in the blood	This information is useful for determining intake and factors that affect intake, transport, activation, etc.
Clinical trials on efficacy and dose response data	Conducted in healthy populations. Bioactive must be measured. Accepted endpoint linked to decreased risk of the particular disease. If surrogate marker, must be “accepted” by regulatory agencies	Need dose response data to determine the efficacious level, and determine intake values
Safety data at the level of intake that might be anticipated	Ideally would include safety data for special populations such as children, pregnant or lactating women	Need this information even if the bioactive is considered generally regarded as safe (GRAS). GRAS means “safe for intended use”
Systematic reviews and/or meta-analyses showing efficacy	In the US, the Institute of Medicine now requires systematic reviews for setting DRI values (most recent was calcium and vitamin D). The US Dietary Guidelines now requires these also	Having a systematic review that shows efficacy is a real plus and may be necessary, e.g., a Cochrane review. These reinforce the need to have major prospective epidemiological studies and randomized clinical trials
A plausible biological explanation for efficacy	This is not required but is a very large plus if it is available	Scientists/evaluators of the research are more comfortable if there is an explanation, particularly if that explanation is accepted by the scientific community

A consensus arose from this scientific symposium on “Nutrient Reference Values—Non-Communicable Disease Endpoints” that there is a sufficient framework and valid scientific information to begin the process of establishing DRIs (in the US/Canada) and NRV-NCDs under the auspices of Codex Alimentarius. As noted above, such a framework and the setting of numerical DRI/NRV-NCD values would be a benefit to scientists working in this field, to funders of the research, to governments, and to consumers. Such reference values would also stimulate innovation in the development of new functional foods and dietary supplements.

The goal of science is to seek truths that can be replicated. Trying to do something again and again and expecting a different outcome is linked to the definition of insanity. In a world with limited resources, and serious health and demographic challenges, if nutrients are to be considered beneficial, the scientific community needs to start asking more logical and direct questions linking observational data to mechanisms of action. Epidemiology provides clues, investigative science needs to provide hypotheses, and robust studies need to be targeted for outcomes that may be far different than that expected in pharmaceutical evaluations. To that end, we need evidence-based science to come to the rescue of this apparent dichotomy of reason within our scientific community.

Designing studies to look at endpoints based on the pharmaceutical model of efficacy when applied to dietary nutrients seems to be a difficult undertaking. First, in the area of nutritional intake or supplementation we are dealing with largely healthy populations that are not demonstrating significant disease states, whereas in clinical evaluations of novel pharmaceutical agents the study subjects by definition should be demonstrating a disease state which the novel drug is attempting to mediate or remedy. In the case of nutrients taken over long periods of time in relatively healthy populations, a different paradigm of investigation per se is required. The considerations of length of time a substance is ingested, the statistical evidence of absence of disease in these populations over time, the hypotheses of metabolism and protective value of these nutrients are all parts of a new dynamic framework of investigative science. During the symposium in Germany, a very enlightening evaluation of the myriad of studies conducted on omega-3 compounds indicated that methodologies of administration might have need of adjustment in order to appropriately consider the findings as representative of value in supplementation of these compounds.
